# l-Methionine repressible promoters for tuneable gene expression in *Trichoderma reesei*

**DOI:** 10.1186/s12934-015-0308-3

**Published:** 2015-08-14

**Authors:** Robert H. Bischof, Jennifer Horejs, Benjamin Metz, Christian Gamauf, Christian P Kubicek, Bernhard Seiboth

**Affiliations:** Austrian Centre of Industrial Biotechnology (ACIB) GmbH c/o Institute of Chemical Engineering, Technische Universität Wien, Gumpendorferstraße 1a, 1060 Vienna, Austria; Biotech and Renewables Center, Clariant GmbH, 81477 Munich, Germany; Research Division Biotechnology and Microbiology, Institute of Chemical Engineering, Technische Universität Wien, Gumpendorferstraße 1a, 1060 Vienna, Austria; Vogelbusch Biocommodities GmbH, Blechturmgasse 11, 1051 Vienna, Austria

**Keywords:** *Trichoderma reesei*, Promoter, Repressible promoter, Tuneable promoter, TauD/TfdA like dioxygenase, l-Methionine, Wheat straw

## Abstract

**Background:**

*Trichoderma reesei* is the main producer of lignocellulolytic enzymes that are required for plant biomass hydrolysis in the biorefinery industry. Although the molecular toolbox for *T. reesei* is already well developed, repressible promoters for strain engineering and functional genomics studies are still lacking. One such promoter that is widely employed for yeasts is that of the l-methionine repressible *MET3* gene, encoding ATP sulphurylase.

**Results:**

We show that the *MET3* system can only be applied for *T. reesei* when the cellulase inducing carbon source lactose is used but not when wheat straw, a relevant lignocellulosic substrate for enzyme production, is employed. We therefore performed a transcriptomic screen for genes that are l-methionine repressible in a wheat straw culture. This analysis retrieved 50 differentially regulated genes of which 33 were downregulated. Among these, genes encoding transport proteins as well as iron containing DszA like monooxygenases and TauD like dioxygenases were strongly overrepresented. We show that the promoter region of one of these dioxygenases can be used for the strongly repressible expression of the *Aspergillus niger sucA* encoded extracellular invertase in *T. reesei* wheat straw cultures. This system is also portable to other carbon sources including d-glucose and glycerol as demonstrated by the repressible expression of the *Escherichia coli lacZ* encoded ß-galactosidase in *T. reesei*.

**Conclusion:**

We describe a novel, versatile set of promoters for *T. reesei* that can be used to drive recombinant gene expression in wheat straw cultures at different expression strengths and in an l-methionine repressible manner. The dioxygenase promoter that we studied in detail is furthermore compatible with different carbon sources and therefore applicable for manipulating protein production as well as functional genomics with *T. reesei*.

**Electronic supplementary material:**

The online version of this article (doi:10.1186/s12934-015-0308-3) contains supplementary material, which is available to authorized users.

## Background

*Trichoderma reesei* is a widely employed microorganism for the production of technical enzymes, including cellulases and hemicellulases that are used by a variety of industries including the biorefinery industry [1–3]. As a result, a relatively well developed toolbox for the genetic engineering of *T. reesei* has become available over the past years [4]. Tuneable promoters are essential tools in this respect and represent a versatile means to alter gene expression at the level of gene transcription [5]. Repressible promoters in particular are useful because (1) they allow the conditional switch-off of genes when e.g. the permanent deletion of that gene is lethal; and (2) they allow the transient control of metabolic pathways, e.g. to slow down metabolism of an intracellular substance leading to its accumulation. Both strategies are equally important for strain construction of industrial producer strains as well as functional genomics studies. In the context of industrial enzyme production, the use of an inexpensive repressing substance that is effective at low concentrations is mandatory. However, a repressible promoter meeting these specifications is currently not available for *T. reesei*, where tuneable expression is almost exclusively controlled by the promoters of the cellobiohydrolase *cel7a* or endoxylanase *xyn1* [4]. In these two cases, the repression is mediated by d-glucose [6, 7], which in turn leads not only to the repression of cellulases and xylanases but also to the repression of most of the other carbohydrate active enzymes (CAZYmes) [8, 9] which is usually undesirable. Repressible expression systems that have been proven to work in filamentous fungi and yeast include the thiamine repressible *thiA* system of *Aspergillus fumigatus* and *Aspergillus oryzae* [10, 11] and the copper dependent *Saccharomyces cerevisiaeCTR4* system in *Schizosaccharomyces pombe* and *Cryptococcus neoformans* [12–14]. Both systems have the advantage of responding to µM concentrations of the respective modulating substances, but suffer from the fact that these compounds may already be present in industrially used complex carbon sources such as wheat straw. Another widely employed system for different yeasts including *S. cerevisiae*, *Ashbya gossypii*, *Candida albicans* or *Pichia pastoris* is the l-methionine repressible *MET3* system [15–18], which however has not been tested in filamentous fungi so far. We regard l-methionine as a promising repressor because its addition to *T. reesei* cultures was previously shown to have no effect on growth and CAZyme expression [19]. Owing to its use as a food and feed additive [20] it is also modestly prized and abundantly available. However, we show here that the expression of the *T. reeseimet3* orthologue responds to l-methionine in the same way as the *S. cerevisiae* gene only when lactose, but not when a complex carbon source such as wheat straw is used as the carbon source. Since wheat straw is one of the most relevant feedstocks for biorefineries [9, 21], we developed a repressible expression system for this carbon source. Using a transcriptomic approach, we identified a novel set of l-methionine repressible genes that feature a diverse range of basal expression strengths and degrees of achievable repression. We also demonstrate the applicability of the promoter region of the most strongly expressed of said genes for tightly repressible expression of intra- and extracellular reporter genes on wheat straw as well as on d-glucose and glycerol containing media.

## Results

### Repression of the *T. reeseimet3* (ATP sulphurylase-encoding) gene by l-methionine

**Fig. 1:**
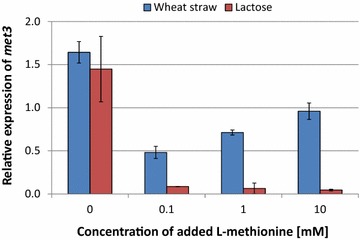
Transcriptional regulation of the *T. reesei met3* following l-methionine addition. l-methionine (0–10 mM) was added to 48 h old wheat straw (*blue bars*) or 40 h old lactose (*red bars*) grown cultures of *T. reesei* and incubated for further 2.5 h before transcript levels were determined. The *met3* transcript levels are related to the expression before addition of l-methionine on wheat straw and lactose, respectively and normalised on *tef1*. Values shown are the mean and standard deviation of three biological replicates from independent cultivations.

The *MET3* encoded ATP sulphurylase is involved in the assimilation of sulphate as part of l-methionine biosynthesis and its promoter has been successfully used in several yeasts to drive tightly repressible expression [15–18]. The *T. reesei* orthologue of the *S. cerevisiaeMET3* gene as well as the orthologues of the other sulphate assimilation and l-methionine biosynthesis genes were analyzed and their encoded activities as well as the gene IDs are shown in the additional files (see Additional file 1: Table S1; Figure S1). When we mapped the individual genes of the pathway to the recently published reconstructed chromosomes of *T. reesei* [22] to test if the genes are clustered, we found a random distribution across all seven chromosomes (see Additional file 1: Table S1). A blastp search with the *S. cerevisiae* Met3 protein against the *T. reesei* filtered protein models retrieved only a single protein with very high sequence similarity (Trire2:47066, E-value of 2.88 × 10^−156^). We therefore tested the expression of the *T. reesei met3* in response to the addition of different concentrations of l-methionine under two different cellulase inducing conditions. Quantitative PCR (qPCR) analysis of *met3* gene expression showed, that it was only partially downregulated by l-methionine when pretreated wheat straw was used as carbon source even at 10 mM of added l-methionine. A stronger repression was monitored when lactose was used as cellulase inducing carbon source. Here, the amount of the *met3* transcript was reduced to about 10 % of the unrepressed control (Fig. 1). We therefore conclude that the *met3* promoter of *T. reesei* cannot be used to tightly shut off gene expression when pretreated wheat straw is used as carbon source.

### Genome-wide identification of l-methionine repressible genes

**Fig. 2:**
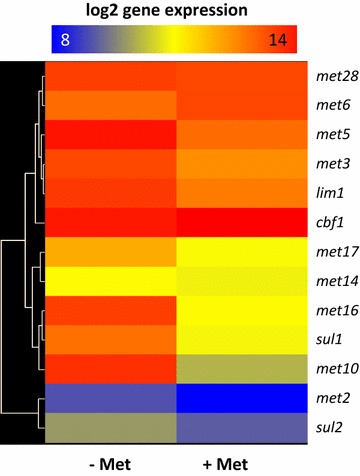
Regulation of l-methionine biosynthesis genes in *T. reesei* QM9414. Heat map showing the regulation of l-methionine biosynthesis genes in the presence (+Met) or absence (−Met) of l-methionine. 0.1 mM l-methionine was added to a 48 h old wheat straw culture of *T. reesei* QM9414 and the transcriptome analysis was performed 2.5 h after l-methionine addition.

In order to identify *T. reesei* genes that are differentially regulated by the addition of l-methionine to the fermentation medium, we compared the transcriptome of *T. reesei* growing on wheat straw in the absence and in the presence of exogenously added 0.1 mM of the amino acid (see “Methods”). As previously shown, these conditions represent a mycelium in the late growth phase [9]. This comparison retrieved 50 genes that were >twofold differentially expressed 2.5 h after the addition of 0.1 mM l-methionine to a 48 h old wheat straw culture (Table 1). 33 of them were downregulated and are thus either directly or indirectly repressed by l-methionine. Four of these genes (*met5*, *met16*, *sul1* and *met2*) encode proteins that are involved in sulfur assimilation and l-methionine biosynthesis. The expression of other genes of these pathways was only slightly reduced in the presence of 0.1 mM l-methionine, and that of *met6* (encoding l-methionine synthase) even slightly enhanced (Fig. 2). The transcription of the *lim1* gene, which encodes the *T. reesei* orthologue of the *S. cerevisiae/N. crassa* F-box protein Met30/SCON-2 [19] was not regulated under these conditions. Other known regulators of l-methionine biosynthesis in *S. cerevisiae* (*MET31*, *MET32*, *MET4*, *MET28* and *CBF1*) either had no or just a very weakly conserved orthologue in *T. reesei* (see Additional file 1: Table S1) and in the latter case they were also not regulated (see Fig. 2). Interestingly, 7 of the 10 l-methionine biosynthesis genes of *T. reesei* also lacked a CACGTG consensus sequence that is bound in *S. cerevisiae* by the Cbf1 protein and involved in the regulation of *MET* genes [23, 24]. When balancing the genes repressed by the addition of 0.1 mM l-methionine to a wheat straw culture, it is intriguing to note that more than a third (13 of 37) of them encode proteins either involved in iron acquisition (iron transporter, ferrooxidoreductase, siderophore biosynthesis, oxalate homeostasis) or contain iron as an essential part for catalysis (flavin- and DszA-type monooxygenases, α-ketoglutarate dependent TauD like dioxygenases; Table 1). Also, the l-methionine repressible transcriptome was overrepresented (p = 0.007) by nine genes encoding transporters for amino acids and other nitrogen-containing compounds. These included three amino acid transporters of which Trire2:60144 shows a high similarity to high affinity l-methionine transporters, as well as allantoine-like transporters that could be involved in transport of pyrimidine bases or oligopeptides [25, 26] and Na^+^-coupled amino acid transporters. Unlike in other transcriptomic studies with *T. reesei* [9, 27, 28], only four of the downregulated genes (=8 % of the regulated transcriptome) comprised unknown genes, and no orphan genes were detected. As expected, none of the major plant biomass degradation related CAZymes were differentially regulated by the addition of 0.1 mM l-methionine which is in perfect agreement with the findings reported by Gremel et al. [19].

**Table 1:** l-Methionine regulated genes of wheat straw grown *T. reesei* QM9414 cultures

Gene ID	Fold change		Log2 gene expression from array data		Functional annotation
			Met−	Met+	
112567	20.02	Down	12.49	8.16	Taurine catabolism dioxygenase TauD/TfdA^a^
6005	11.61	Down	11.55	8.01	MSF permease, allantoin-permease like^c^
54461	8.98	Down	10.09	6.92	MSF permease^c^
73250	8.76	Down	10.30	7.16	Urea transporter/Na^+^-amino acid transporter^c^
59876	8.61	Down	12.02	8.91	DszA-type family xenobiotic monooxygenases^a^
104081	8.55	Down	10.63	7.53	DszA-type family xenobiotic monooxygenases^a^
60144	8.54	Down	10.54	7.45	Amino acid transporter, high affinity methionine transporter^c^
69563	8.08	Down	9.79	6.77	MSF permease^c^
123979	6.61	Down	12.75	10.03	Taurine catabolism dioxygenase TauD/TfdA^a^
5369	5.83	Down	9.40	6.86	Metallocarboxypeptidase, putative
59333	5.61	Down	9.12	6.63	MSF permease, allantoin permease like^c^
56911	5.59	Down	9.89	7.41	Urea transporter/Na^+^ amino acid transporter^c^
103039	5.06	Down	11.34	9.00	Peptidase S41
81576	5.04	Down	13.64	11.31	Assimilatory sulfite reductase, alpha subunit^b^
103012	4.75	Down	12.35	10.10	Taurine catabolism dioxygenase TauD/TfdA^a^
64049	4.48	Down	10.47	8.31	Unknown protein
62872	3.88	Down	14.05	12.10	Unknown protein GPR1/FUN34/yaaH-like
68831	3.81	Down	10.63	8.70	Amino acid transporter^c^
65410	2.95	Down	13.51	11.95	Phosphoadenosine phosphosulfate reductase^b^
124115	2.93	Down	13.34	11.79	Phosphoenolpyruvate carboxykinase AcuF
22110	2.79	Down	11.54	10.06	Flavin-containing monooxygenase^a^
53964	2.56	Down	8.68	7.33	Oxalate decarboxylase^a^
109239	2.56	Down	9.31	7.95	Unknown protein
108781	2.48	Down	10.93	9.62	DszA-type family xenobiotic monooxygenases
121139	2.39	Down	11.10	9.84	Amino acid transporter, AAT-family^c^
79741	2.38	Down	13.11	11.86	Sulfat transporter SUL1^b^
69696	2.36	Down	10.18	8.94	DszA-type family xenobiotic monooxygenases^a^
111912	2.35	Down	9.36	8.14	Carbonic anhydrase
120473	2.32	Down	11.41	10.19	Dihydrolipoamide transacylase
54219	2.28	Down	14.81	13.61	CE5 acetyl xylan esterase
112590	2.26	Down	9.26	8.09	Siderophore biosynthesis lipase/esterase^a^
102820	2.18	Down	11.17	10.04	Ferrooxidoreductase^a^
71005	2.16	Down	8.35	7.24	NRPS siderophore synthase SID1^a^
66517	2.11	Down	12.63	11.55	Homoserine *O*-acetyltransferase^b^
120864	2.16	Up	10.76	11.88	DIP2, encoding a nucleolar protein
4109	2.26	Up	10.68	11.85	Aspartate/tyrosine/aromatic aminotransferase
78650	2.21	Up	11.92	13.06	C2H2 transcriptional regulator
56587	2.44	Up	8.81	10.10	GCN5-related N-acetyltransferase
41590	2.09	Up	11.58	12.64	Iron transporter^a^
3049	3.46	Up	11.76	13.55	Methionine aminopeptidase
66854	3.14	Up	7.57	9.22	Monocarboxylate transporter
60052	2.25	Up	8.15	9.32	Short chain dehydrogenase/reductase
67840	2.06	Up	10.62	11.66	Shows similarity to *S. cerevisiae* Utp11
110767	2.31	Up	9.70	10.91	Unique protein
120975	3.17	Up	9.57	11.23	Unknown protein
110768	2.44	Up	10.77	12.06	Unknown protein
77593	2.15	Up	9.11	10.21	Unknown protein
56048	3.60	Up	8.96	10.81	Unknown protein
55172	2.06	Up	10.00	11.05	Unknown protein
106043	2.87	Up	9.02	10.55	Unknown protein, GFA-domain
43397	3.33	Up	9.62	11.35	Unknown protein, secreted, only in hypocreaceae, HTG
57494	2.05	Up	9.98	11.00	UTP10, encoding a component of the SSU processome

^a^Iron related proteins.

^b^Sulfur metabolism related proteins.

^c^Transport proteins.

**Fig. 3:**
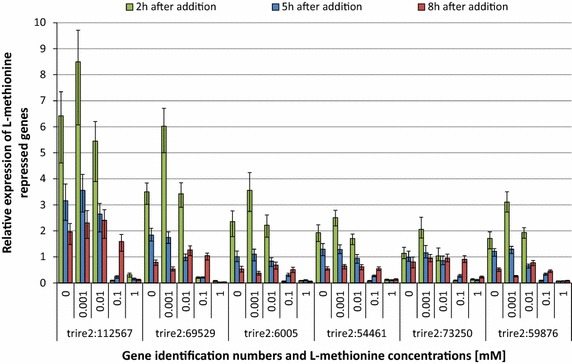
Transcriptional profile of selected l-methionine repressible genes. Transcript levels of the six most highly regulated genes of the transcriptome analysis were tested following the addition of different amounts of l-methionine (0–1 mM) to a 48 h old wheat straw culture of *T. reesei* QM9414. The expression was correlated to that of a sample taken just before the addition of l-methionine and normalized on *tef1.*

The expression of the five most strongly repressed genes was then tested by qPCR over a longer time period and with different concentrations of the repressor. As shown in Fig. 3 repression of these genes by l-methionine was strong 2 and 5 h after its addition at concentrations ≥0.1 mM but not detectable ≤10 µM. 8 h after l-methionine addition, the cultures supplemented with 0.1 mM of the amino acid showed transcript levels of the tested genes similar to the non-treated controls, suggesting that the repression is of a transient nature. In fact, the repression by 0.1 mM l-methionine declined already 5 h after its addition. When a final concentration of 1 mM l-methionine was added, repression of all tested genes was tight at all tested time points.

### Construction of *T. reesei* strains with repressible *lacZ* and *sucA* reporters

We then selected the promoter (subsequently termed P_*123979*_) of the TauD like dioxygenase-encoding gene *trire2*:*123979* for further testing, because it displayed the highest level of transcription of this gene family in the absence of exogenous l-methionine (log2 fluorescence of 12.75) and strong repression in the presence of 0.1 mM added l-methionine (see Table 1). As reporter genes, we chose a modified version of *Aspergillus niger* invertase encoding *sucA* (ß-d-fructofuranosidase, EC 3.2.1.26; [29]) and the *E. coli* ß-galactosidase encoding *lacZ*, respectively. As a comparison to the expression strength of P_*123979*_, we used the strong constitutive promoter of *tef1* (P_*tef1*_) as previously described [30, 31]. Both promoter regions were inserted into *sucA* and *lacZ* expression plasmids that also contained the *hphB* resistance cassette for selection. After transformation of the *sucA* expression cassettes into *T. reesei* QM9414 and purification of the transformants, we isolated at least three positive strains for the repressible promoter and two P_*tef1*_ reference strains having a single integration of the construct as verified by qPCR (see Additional file 1: Figure S2). In the same way, we also constructed strains expressing *E. coli lacZ* under the control of the promoters of P_*123979*_ and P_*tef1*_ and again isolated strains having a single copy integration of the expression cassette (see Additional file 1: Figure S3).

### l-Methionine dependent repression of *sucA* expression on wheat straw medium

**Fig. 4:**
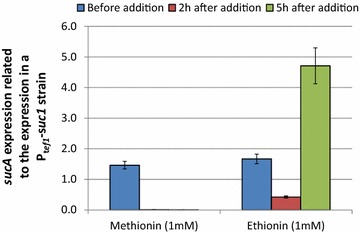
Transcript levels of *sucA* in response to 1 mM l-methionin or l-ethionin. Transcript levels before as well as 2 and 5 h after addition of 1 mM l-methionin or l-ethionin to a 48 h old wheat straw cultures of *T. reesei* strains expressing the reporter gene *sucA* under the control of P_*123979*_. Transcript levels are related to the expression of a *sucA* constitutively expressing *T. reesei* strains (P_*tef1*_-*sucA*) in a 48 h old wheat straw culture and normalised on *tef1*. Values show the mean and standard deviation of two independent strains.

**Fig. 5:**
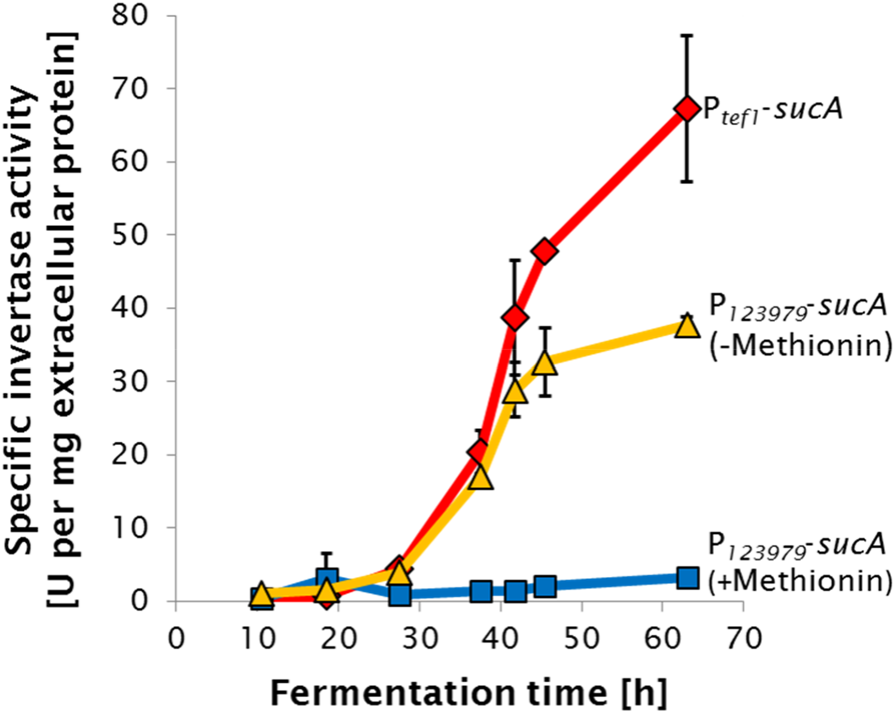
Specific invertase activity in the supernatants of *sucA* expressing *T. reesei* strains. Constitutively *sucA* expressing (*red line*, P_*tef1*_-*sucA*) as well as l-methionine non repressed (*yellow line*, −Met) and repressed (*blue line*, +Met) P_*123979*_ *-sucA* expression strains are shown. Values are the mean and standard deviation from three (P_*123979*_-*sucA*) or two (P_*tef1*_-*sucA*) independent strains.

We then cultured the different *sucA* expressing strains on wheat straw containing medium for 48 h and added l-methionine to a final concentration of 1 mM to the medium. We found that prior to the l-methionine addition the three P_*123979*_ strains accumulated 144 ± 10 % of the *sucA* mRNA as compared to P_*tef1*_ strains, while already 2 and 5 h after the l-methionine addition below 1 % of that amount was detected indicating a strong repression (Fig. 4, left side). The expression level in both P_*tef1*_–*sucA* strains was furthermore indiscernible at a 95 % confidence interval. We then also compared the specific invertase activity in the supernatant of the P_*123979*_ expression strains with that of the P_*tef1*_ reference strains grown on wheat straw containing medium as shown in Fig. 5. When we added l-methionine to a final concentration of 1 mM to the fermentation medium of the P_*123979*_ strains at 18.5 h and another 5 mM at 27.5 h, we found only a drastically reduced accumulation of invertase activity in the supernatant at later time points, indicating that the promoter was tightly shut during that period. When we added the same amount of l-methionine to the P_*tef1*_ strains, we found no difference in the rate of invertase production during the next 18 h (2.46 ± 0.09 units per hour without l-methionine, 2.41 ± 0.09 units per hour with l-methionine). We also monitored the invertase activity in the supernatant of untransformed *T. reesei* QM9414 and found that it was below one unit per mg extracellular protein after 24, 48 and 72 h of fermentation and declined over the course of the fermentation.

### Portability of the l-methionine repressible expression system to other carbon sources and alternative repressors

We then wanted to learn whether the P_*123979*_ promoter region could be used for the repressible expression on a broader range of carbon sources including the cellulase repressing carbon source d-glucose or the cellulase neutral carbon source glycerol, both of which are also more relevant in terms of functional genetics than wheat straw is. To this end, we tested the repression of the intracellularly produced ß-galactosidase LacZ in two of the P_*123979*_-*lacZ* strains during growth on minimal medium containing d-glucose or glycerol as carbon sources. To account for the different growth rates on both media the strains were cultured for 16 or 30 h, respectively and l-methionine added to a final concentration of 1 or 5 mM. 10 h later, mycelia were harvested and the intracellular LacZ activity determined. As shown in Table 2, both l-methionine concentrations (1 and 5 mM) led to a tight repression of P_*123979*_ as compared to the control experiment.

**Table 2:** Repression of LacZ activity in *T. reesei* d-glucose and glycerol cultures

	Basal	l-Methionine		l-Cysteine	
		1 mM	5 mM	1 mM	5 mM
Glucose					
P_*123979*_-*lacZ* 3	1.27 ± 0.15	0.04 ± 0.01	0.00	1.02 ± 0.07	0.00
P_*123979*_-*lacZ* 7	1.46 ± 0.12	0.05 ± 0.00	0.00	0.81 ± 0.31	0.00
QM9414	0.00				
Glycerol					
P_*123979*_-*lacZ* 3	1.09 ± 0.08	0.09 ± 0.02	0.03 ± 0.00	n.d	n.d
P_*123979*_-*lacZ* 7	1.02 ± 0.59	0.10 ± 0.00	0.00	n.d	n.d
QM9414	0.05 ± 0.00				

Intracellular LacZ activity relative to an equally treated constitutively expressing lacZ strain (P_*tef1*_-*lacZ*).

n.d. activity not determined.

l-cysteine is often used as an alternative repressor for *MET3* orthologues in different yeasts and is linked to l-methionine metabolism via homocysteine and cystathionine (Additional file 1: Figure S1). When we tested the effect of l-cysteine on P_*123979*_ repression, only the 5 mM concentration had an equally pronounced effect, whereas concentrations of 1 mM resulted only in a minor reduction of ß-galactosidase activity. Since repression by lower concentrations of l-methionine appeared as a transient phenomenon in this study, we also tested the effect of the non-metabolizable substitute l-ethionine on two of the P_*123979*_–*sucA* strains. However, as shown in Fig. 4, l-ethionine addition failed to reproduce the tight repression observed with l-methionine 2 h after its addition and about a third of the initial *sucA* mRNA could still be detected. After 5 h, the transcript level even surpassed the basal level before l-ethionin addition more than threefold which shows that l-ethionin cannot be used for repression of P_*123979*_.

## Discussion

l-methionine as a repressor of gene expression in yeasts is well established. In *S. cerevisiae*, repression of the l-methionine biosynthesis genes *MET2*, *MET3*, *MET5*, *MET6*, *MET14 and MET25* has been shown [17, 32] and the promoters of the *MET3* orthologues of *C. albicans*, *A. gossypii* and *P. pastoris* have been used for repressible gene expression [15, 16, 18]. Our results show that when a complex carbon source such as pretreated wheat straw is used, only the phosphoadenylyl-sulfate reductase encoding *met16* (*trire2*:*65410*), sulfite reductase encoding *met5/ecm17* (*trire2*:*81576*), homoserine O-acetyltransferase encoding *met2* (*trire2*:*66517*) and sulfate permease encoding *sul1* (*trire2*:*79741*) are more than twofold repressed by 0.1 mM l-methionine in *T. reesei*. In contrast, the orthologue of sulfate adenylyltransferase encoding *met3* (*trire2*:*47066*) was strongly repressible by l-methionine at concentrations higher than 0.1 mM only when lactose was used as the carbon source and the degree of repression was comparable to that observed in *S. cerevisiae* [17, 32].

To identify novel repressible promoters we used therefore a genome-wide screen under l-methionine repressing conditions. We found genes for transport proteins to be a highly enriched group in the downregulated transcriptome of *T. reesei* confronted with l-methionine. Two of the transporters that were downregulated in the presence of l-methionine appear to be high affinity amino acid transporters and one of them indeed shows moderate sequence similarity to the characterized *S. cerevisiae*l-methionine and l-cysteine transporter Mup1 ([33], blastp score 4.7 E^−78^). It is therefore tempting to speculate that the uptake of l-methionine itself represents another control circuit for the maintenance of l-methionine homeostasis in *T. reesei*. Repression of l-methionine transport by l-methionine at the transcriptional level also occurs in *Penicillium chrysogenum* and *S. cerevisiae* [34, 35] as well as in *E. coli* [36]. Our findings that a third of the genes that were repressed by 0.1 mM l-methionine encoded proteins involved in iron acquisition or contained iron suggested a relationship between iron metabolism and l-methionine availability. Recently, Amich et al. [37] have demonstrated a cross-talk between iron homeostasis and sulphur metabolism: starving an *Aspergillus fumigatus* mutant defective in the sulfur metabolite transcriptional repressor MetR for sulphur results in increased expression of the genes of the iron regulon. The molecular basis for this cross-talk is the upregulation of iron acquiring proteins when the biosynthesis of Fe-S cluster enzymes is impaired because of a shortage of l-cysteine [38]. Consequently, loss-of-function of MetR—and thus sulfur derepression—results in increased cellular iron as well as ferricrocin contents accompanied by transcriptional derepression of genes involved in iron acquisition such as siderophore biosynthesis and uptake as well as reductive iron assimilation [37]. Our transcriptomic results under l-methionine addition (=sulfur repression) are essentially the opposite of those with the *A. fumigatus* MetR mutant and would therefore be in perfect agreement with the operation of an iron-sulfur crosstalk also in *T. reesei*. However, the strong repression of the iron containing oxygenases may also have an alternative explanation: in bacteria, Fe^2+^/α-ketoglutarate dependent TauD/TfdA like dioxygenases are involved in the release of sulfur from the aminosulfonic acid taurine and other organic sources of sulfur [39]. In *E. coli*, their transcription as well as that of other sulfur starvation induced genes is positively influenced by the transcription factor Cb1 [40–42]. A negatively regulating element in this system is the sulfur assimilation metabolite adenosine 5′-phosphosulfonate, which binds Cb1 and abolishes its activating function [43, 44]. Similarly, dibenzothiophene sulfone monooxygenase (DszA) is involved in biodesulfurization in *Rhodococcus erythropolis* [45, 46] and repressed by both l-methionine and l-cysteine. If a similar regulation occurs in fungi, it would offer an alternative explanation for the negative regulation of these oxygenases by l-methionine. This in turn would mean that this system can likely also be transferred to other species, because TauD/TfdA like dioxygenases are in fact quite common in other *Pezizomycotina* and particularly also among industrially relevant *Aspergillus* spp. and the plant pathogenic *Fusarium* spp. (Additional file 1: Figure S4). Although the transient nature of l-methionine repression of the P_*123979*_ promoter at low concentrations is advantageous to effectively deactivate and reactivate gene expression, it might also be useful to have a repression system that is tightly shut by low concentrations of a repressor during a longer period of time. Attempts to use the l-methionine non-metabolizable analogue l-ethionine for this purpose failed, which probably points to the fact that—like in *S. cerevisiae* and *A. nidulans* [47, 48]—S-adenosylmethionine, rather than l-methionine itself, is the repressing metabolite. l-Ethionine cannot be adenylated and inhibits the SAM-dependent methyltransferases [49]. An alternative to overcome this bottleneck would be to knock out the gene further metabolizing S-adenosylmethionine (i.e. adenosyl-homocysteine hydrolase) or placing it under the control of an l-methionine repressible promoter.

## Conclusions

In the present manuscript we describe novel l-methionine repressible genes that serve as an alternative to the well-known *S. cerevisiaeMET3* system under conditions where its *T. reesei* orthologue *met3* is non-repressible, i.e. when the industrially relevant carbon source wheat straw is used. The described set of repressible genes covers a wide array of expression strengths and levels of achievable repression. We demonstrate that we can use the promoter region of a TauD/TfdA-like dioxygenase to achieve repressible gene expression of two reporter genes in *T. reesei* and that this system can also be used on less complex carbon sources such as d-glucose or glycerol and is likely transferable also to other fungi. Taken together these findings show that the present system offers a versatile means to realize repressible gene expression and therefore represents a valuable tool for industrial strain engineering and functional genetics of *T. reesei*.

## Methods

### Strains and culture conditions

*Trichoderma reesei* QM9414 (ATCC 26921) was used throughout the study and maintained on potato dextrose agar (Difco). Cultivation of *T. reesei* with pretreated wheat straw, d-glucose, glycerol or lactose as the sole carbon source was routinely performed as previously reported [9]. In brief, pretreated wheat straw up to a final concentration of 10 g L^−1^ dry weight was added to unbuffered mineral medium, the pH set to 4.8 with 1 M KOH and then autoclaved. CaCl_2_ was added separately after autoclaving as were the other carbon sources. 1 × 10^6^ spores ml^−1^ were then aseptically added to 200 ml of the medium in a 1 L Erlenmeyer flask. The culturing conditions were 28 °C and 250 rpm in a rotary shaker. For the cultivations where extracellular invertase activity was measured, a slightly modified medium that contained per liter: 5 g (NH_4_)_2_SO_4_, 2 g KH_2_PO_4_, 0.3 g MgSO_4_, 0.4 g CaCl_2_, 1.7 g yeast nitrogen base without amino acids and (NH_4_)_2_SO_4_, 10.22 g (i.e. 50 mM) potassium phthalate, 20 g pretreated wheat straw, 5 mg FeSO_4_ × 7H_2_O, 1.6 mg MnSO_4_ × H_2_O, 1.4 mg ZnSO_4_ × 7 H_2_O, 1.61 mg CoCl_2_ and 0.1 mg CuSO_4_ × 5 H_2_O was used.

### Transcriptomic analysis

*Trichoderma reesei* QM9414 cultures were pregrown for 48 h on wheat straw medium after which 0.1 mM of l-methionine were added to the cultures. Mycelia were harvested after 2.5 h, washed and instantly frozen in liquid nitrogen. Genome wide gene expression analysis was performed as previously described [9]. The microarray data and the related protocols are available at the GEO web site (http://www.ncbi.nlm.nih.gov/geo/) under accession number: GSE69745. Groups of eukaryotic orthologous genes (KOG) were retrieved from the *T. reesei* genome website (http://genome.jgi-psf.org/cgi-bin/kogBrowser?db=Trire2). When no KOG assignment was available there, sequences were manually assigned after sequence analysis using Interpro (http://www.ebi.ac.uk/interpro/) and OrthoDB (http://orthodb.org/orthodb7). In ambiguous cases, KOG category S for “unknown function” was assigned. Enrichment of KOG groups was determined by Fisher’s exact test.

### Analysis of the l-methionine biosynthesis pathway and related pathways in *T. reesei*

Protein sequences were retrieved from the KEGG catalogue of the JGI *T. reesei* portal (http://genome.jgi-psf.org/cgi-bin/metapathways?db=Trire2) and verified by comparison to the NCBI database using blastp.

### Reverse transcription qPCR

Culture conditions were identical to the cultures from the transcriptome analysis if not stated otherwise and methods for total RNA extraction and qPCR measurements are described in detail elsewhere [9]. Primers sequences and amplification efficiencies are given in the supplementary material (Additional file 1: Table S2). Determination of the PCR efficiency was performed using triplicate reactions from a decadic dilution series of cDNA, and the amplification efficiency then calculated from the given slopes using the realplex v2.2 software. Expression ratios were calculated using REST© software [50].

### Construction of *sucA* and *lacZ* reporter vectors

The *sucA* reporter gene was *de novo* synthesized by MWG Operon (Ebersberg, Germany), whereby a 6x His-tag was introduced C-terminally and the native SucA signal peptide was replaced by the signal peptide from *T. reesei* CEL7A (MYRKLAVISAFLATARA). Also, two restriction sites (*Sal*I and *Cla*I) of the *sucA* ORF were removed by silent mutations (Codon526:CAC⇒GAC; Codon541:AAT⇒GAT). Constructions were made in pBS_*gfp*_T*hH3*_*hph*, an *egfp* expression vector consisting of the *egfp* gene, a 369 bp terminator fragment of the histone 3 (*hH3*) terminator and the *hph* expression cassette [51] as selection marker (Robert Bischof and Bernhard Seiboth, unpublished data). The *sucA* gene was inserted into the *Cla*I/*Sal*I fragment of pBS_*gfp*_T*hH3*_*hph* replacing *egfp* and resulting in pRB-X. Fragments of the candidate l-methionine repressible promoter P_123979_ and the *tef1* promoter region were amplified from chromosomal DNA of *T. reesei* QM9414 using primers Inf_pRB-XVI_fw and Inf_pRB-XVI_rv and Inf_pRB-XVIII_fw and Inf_pRB-XVIII_rv, respectively and subsequently introduced into the *Sal*I site of pRB-X by recombinational cloning with the Infusion^®^HD Cloning Kit (ClonTech, CA, USA) resulting in plasmids pRB-XVI (P_*123979*_-*sucA*) and pRB-XVIII (P_*tef1*_-*sucA*), respectively. Similarly, the *lacZ* gene (GenBank:NC_000913) was *de novo* synthesized, the *Cla*I site removed (Codon 280 ATC⇒ATT) and inserted into the *Sal*I/*Cla*I site of pBS_*gfp*_T*hH3*_*hph* resulting in pRB-XI. For the construction of *lacZ* reporter vectors, the *lacZ* β-complementation region of pLHhph [51] was eliminated by PCR amplification of the vector using primers hphF and pLHhph-lacZ_rv. The PCR mix was then digested with DpnI to remove the template DNA by methylation specific restriction. The promoter fragments of *trire2*:*123979* (i.e. P_*123979*_) and *tef1* (P_*tef1*_) were amplified from chromosomal *T. reesei* DNA using primers XVII_inf_P_fw and XVII_inf_P_rv and XIX_inf_P_fw and XIX_inf_P_rv, respectively. The *lacZ* gene was amplified from pRB-XI using primers XVII_inf_L_fw and XVII_inf_L_rv (for P_*123979*_) and XIX_inf_L_fw and XIX_inf_L_rv (for P_*123979*_), respectively. Both fragments were simultaneously inserted into the pLHhph-*lacZ* DNA fragment via Infusion^®^HD Cloning Kit resulting in pJH-III (P_*123979*_-*lacZ*) and pJH-IV (P_*tef1*_-*lacZ*). Oligonucleotides are given in the supplementary material (Additional file 1: Table S2).

### Fungal transformation and strain characterization

Recombinant *T. reesei* strains were generated by protoplast mediated transformation as described earlier [52] using MEX plates containing 100 µg ml^−1^ hygromycin for selection. Transformants were then passaged to PDA plates containing the same amount of hygromycin and subsequently purified twice via single spore isolation on PDA plates containing 0.1 % (w/v) Triton X-100 as colony restrictor. DNA extraction, PCR screening and gene copy number estimation by qPCR was done essentially as described by Tisch et al. [53]. Primer sequences for the PCR screening are given in the supplementary material (Additional file 1: Table S2) and positive (plasmid DNA) as well as negative controls (genomic DNA of *T. reesei* QM9414) were included in the PCR screening.

## Measurement of extracellular invertase activity

5 ml culture broth samples were prefiltered through Miracloth tissue and centrifuged. Supernatants were immediately snap-frozen in liquid nitrogen and kept on −20 °C until they were analyzed. Invertase activity towards 12.5 mM saccharose in the supernatant was assayed in 50 mM sodium citrate buffer (pH = 4.6) at 25 °C for 20 min and the reaction stopped by the addition of 50 mM Trizma base. Each sample was assayed as a technical triplicate. The released d-glucose was assayed using a commercially available d-glucose assay reagent (Sigma, St. Louis, MO, USA). Protein concentrations were determined with the Bio-Rad protein assay using BSA as standard (Biorad, Hercules, CA, USA). Non inoculated substrate controls were included in both cases and the absorbances read out using a Spectra Max Plus 384 microplate reader (Molecular Devices, Sunny Vale, CA, USA). Coefficients of variation were typically at or below 5 %. One unit was defined as the amount of enzyme, which hydrolyses 1 µmol of sucrose to d-glucose and d-fructose per minute.

### Measurement of intracellular ß-galactosidase activity

The mycelium from 10 ml of culture broth was harvested by filtration with Miracloth tissue, washed with 50 ml ice cold tap water and snap-frozen in liquid nitrogen. Mycelia were stored at −80 °C until analyzed. Cell extracts were prepared as principally described elsewhere [54] but using EDTA free cOmplete ULTRA Tablets (Roche, Dubai, UAE) instead of phenylmethylsulfonfluorid and pepstatin A only. ß-galactosidase activity towards 20 mM ortho-nitrophenyl-ß-d-galactosid (ONPG) was assayed in 10 mM phosphate buffer (pH = 7.4) at 40 °C for 20 min. Each sample was assayed as a biological and technical triplicate. The nitrophenol content of the hydrolysis reaction was directly read out using a Spectra Max Plus 384 microplate reader and the activity calculated using Lambert–Beer’s law and a molar extinction coefficient of 18.5 ml mol^−1^ cm^−1^ for nitrophenol. The intracellular protein content was assayed as described above. One unit was defined as the amount of enzyme, which hydrolyses 1 µmol of ONPG to ß-d-galactose and nitrophenol per minute.

## Additional file

**Additional file 1:** **Table S1.** *T. reesei* orthologues of l-methionine biosynthesis related proteins and genomic location of the corresponding gene; **Table S2.** Oligonucleotides used in this study; **Figure S1.** Metabolic pathway of sulphate assimilation and l-methionine biosynthesis; **Figure S2.** Gene copy number estimation of *T. reesei sucA* transformants by qPCR; **Figure S3.** Gene copy number estimation of *T. reesei lacZ* transformants by qPCR; **Figure S4.** Phylogenetic analysis of TauD like dioxygenases.
